# Generation and characterization of BAC transgenic mouse lines expressing a fluorescent protein in trigeminal and dorsal root ganglion neurons

**DOI:** 10.1371/journal.pone.0321014

**Published:** 2025-06-06

**Authors:** Piu Banerjee, Takuya Sato, Takuji Iwasato

**Affiliations:** 1 Laboratory of Mammalian Neural Circuits, National Institute of Genetics, Mishima, Japan; 2 Graduate Institute for Advanced Studies, SOKENDAI, Mishima, Japan; University Hospital Wurzburg, GERMANY

## Abstract

Genetic tools to identify and isolate specific cell types are required to study different model systems in an organism. Such tools to study sensory neurons in trigeminal and dorsal root ganglia are insufficient, making research progress in somatosensation difficult. Our study aimed to distinctly visualize and identify these peripheral sensory neurons in the mouse somatosensory system. We generated bacterial artificial chromosome transgenic mouse lines expressing the red fluorescent protein (RFP) in nuclei of *Advillin* (*Avil*)-positive neurons to help with the scarcity of bioresources. In these mice, RFP was specifically expressed in the trigeminal ganglion (TG) and dorsal root ganglion (DRG) in adulthood, consistent with previous reports. We also assessed RFP expression in the TG and DRG of these mice during embryonic and postnatal development. The TG RFP expression became evident around embryonic day 16.5 and continued across the postnatal period; in the DRG, it became apparent around postnatal day 0, continuing further. Lastly, almost all TG neurons (~90%) expressed RFP. These transgenic lines are valuable for studying the murine somatosensory system across developmental stages.

## Introduction

The somatosensory system processes tactile sensory inputs, allowing us to feel and respond to external environmental cues and interact with the world around us. Peripheral sensory neurons are vital in transmitting information from the external world to our brain; they are pivotal targets in somatosensation-related studies [1]. These neurons are primarily pseudo-unipolar: a single axon bifurcates into two, with the distal branch innervating the peripheral tissues and the proximal branch innervating the central nervous system brainstem [2,3]. Somata of somatosensory neurons are housed in nodular structures called ganglia in the peripheral nervous system [4]. Those somatosensory ganglia are divided into dorsal root ganglia (DRG) and trigeminal ganglia (TG). The DRG, arranged in a metameric pattern on either side of the spinal cord, have somata of sensory neurons innervating the tissues associated with local body segments (dermatomes). Sacral and lumbar DRG innervate the lower body region, thoracic DRG innervate the trunk, and cervical DRG innervate the forelimbs, shoulder, neck, and head occipital lobe [5,6]. The TG, located on either side of the space on top of the temporal bone, has cell bodies of sensory neurons innervating different head regions (*e.g.,* eye, whisker pad, lower jaw) [7,8]. The peripheral trigeminal nerve is divided into three major branches: the ophthalmic branch, which innervates the supraorbital region (V1); maxillary branch, which innervates the infraorbital region (V2); and the mandibular branch. which innervates the lower jaw region (V3) [9–11]. Within these sensory ganglia, somata are further surrounded by satellite glial cells (SGCs), accounting for a heterogeneous cell population [12–14].

Despite the conspicuous importance of sensory ganglia in research, their visualization and isolation in mice remain challenging, mainly because of their small size and pale color that make it difficult to distinguish them from surrounding tissues, fat, or lymph nodes, especially during early developmental stages [15,16]. Here, we aimed to create a suitable biological resource using appropriate genetics tools to aid with the selective visualization, labeling, and isolation of peripheral sensory neurons housed in the ganglia. To specifically target these cells, we used the promoter of *Advillin* (*Avil*) gene which is almost exclusively expressed in the periphery sensory neurons [8,17–19]. We generated transgenic (Tg) mouse lines expressing the red fluorescent protein (RFP) in the nuclei of *Avil-*positive neurons by using bacterial artificial chromosome (BAC) recombination [20,21]. We characterized the strength and specificity of the RFP signal in the TG and DRG across varying time points from the embryonic to the adult stage. Our analysis allowed us to select two suitable Tg lines: Tg3 and Tg4. An example of the application of the Tg3 line has been reported [15]. In the current study, we describe the generation and characterization of the Avil-nlsRFP Tg3 and Tg4 lines. These mouse lines will assist researchers in visualizing and identifying peripheral sensory neurons across embryonic and postnatal stages.

## Results & discussion

**Fig 1:**
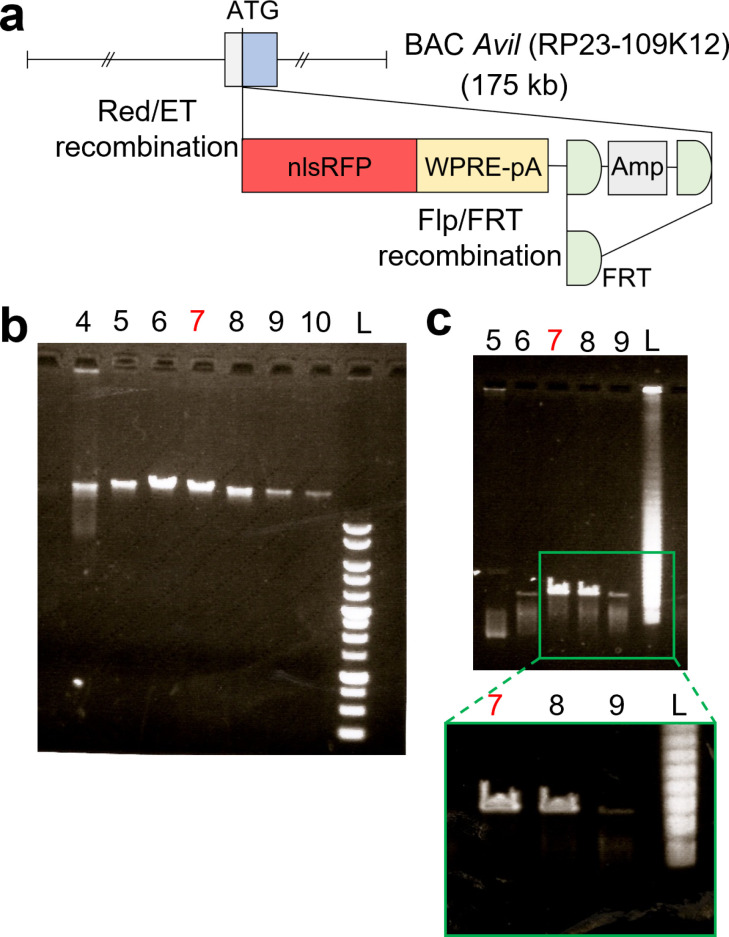
Generation of *Avil-*nlsRFP transgenic mice. (a) Schematic of the *Avil-*nlsRFP BAC transgenic construct generated by Red/ET recombination. The nlsRFP-WPRE-pA- FRT-Amp-FRT cassette was replaced with the initiation codon of the mouse *Avil* gene in the 175-kb BAC clone (RP23-109K12) and subsequently the FRT-Amp-FRT fragment was removed by Flp/FRT recombination. [*Avil: Advillin*, nls: nuclear localization signal, RFP: red fluorescent protein, Amp: Ampicillin]. (b) Sepharose column fractions (#4 to #10) of the linearized BAC construct run on an agarose gel. A 1 kb ladder (L) was used as the size marker. (c) Pulsed-field gel electrophoresis was performed to further distinguish fractions. Fractions #5 to #9 were loaded on 1% agarose gel and ran for 16 h at 5V/cm (Switch time: 50 s to 90 s). Fraction #7 was selected for microinjection into the mouse fertilized eggs. A magnified image of the area enclosed by the green square is shown for better visualization of fragment sizes.

We used the BAC Tg mouse technology to selectively express a fluorescent protein in *Avil-*expressing peripheral sensory neurons in mice. We selected the BAC clone RP23-109K12, which contains the translational initiation site of the mouse *Avil* gene around the center of the clone, and manipulated it by Red/ET-recombination (homologous recombination in *Escherichia coli*) [20–22] to insert a cassette containing the nuclear localization signal (nls) RFP (Fig 1a). The obtained BAC construct was linearized by restriction digestion and purified using a column (Fig 1b). We used pulsed-field gel electrophoresis to choose a fraction that has a desired DNA size (Fig 1c). Fraction #7 was the most appropriate and was microinjected into fertilized mouse eggs, leading to the generation of four founder mice (#1, #2, #3 and #4). Founders #1 and #2 were female, and founders #3 and #4 were male. Founder #2 died before maturation. and the remaining three were backcrossed with wildtype mice to generate their respective Tg lines (Tg1, Tg3, and Tg4). The obtained lines were analyzed by assessing their TGs for RFP fluorescence. TGs isolated from postnatal day 6 (P6) to P8 pups of all three lines exhibited a clear RFP signal (S1 Fig). However, the RFP signal in the TGs of Tg1 pups was much weaker than in Tg3 and Tg4 pups (S1 Fig). Therefore, we did not analyze Tg1 further and selected Tg3 and Tg4 for detailed analyses. The TG of Tg3 pups exhibited a stronger RFP signal than Tg4. Among 36 Tg4 pups, we observed that all 18 males were RFP positive and all 18 females were RFP-negative, indicating that RFP expression in the Tg4 line was linked to the Y chromosome.

**Fig 2:**
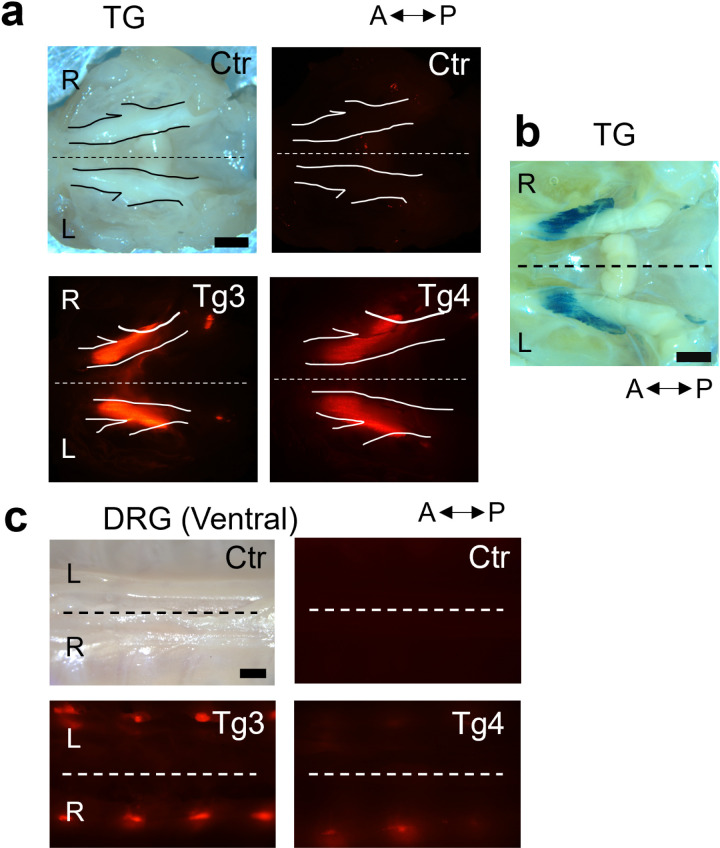
High RFP expression in TG and DRG of *Avil-*nlsRFP mice in adulthood. (a) Top view images of whole trigeminal ganglia (TG) attached to the cranial base. Both Tg3 and Tg4 mice showed strong RFP expression in the TG, with Tg3 considerably brighter than Tg4. Representative brightfield and fluorescent images are shown for wildtype (WT) control (n = 3), Tg3 (n = 3) and Tg4 (n = 3) mice in adulthood. (b) A representative image (top view) of the intact TG attached to the cranial base from adult *Avil-*Cre: Rosa26-loxP-stop-loxP-nlsLacZ (*Avil-*Cre:RNZ) mice. The distribution of sensory neurons is visualized by X-gal staining (n = 2 mice). (c) Images of whole dorsal root ganglia (DRG) embedded along the spinal cord (ventral view). Tg3 mice showed strong RFP expression in the DRG, while the RFP signal from Tg4 DRG was considerably weaker. Representative brightfield and fluorescent images are shown for control (n = 3), Tg3 (n = 3) and Tg4 (n = 3) mice in adulthood. A: Anterior, P: Posterior, R: Right, L: Left, Ctr: Control, dashed lines represent the midline, solid lines outline the TGs. Scale bars: 1 mm.

We isolated entire TGs from *Avil-*nlsRFP Tg3 and Tg4 mice (> P45) and their wildtype littermates to evaluate RFP expression in adulthood. The TG of Tg3 and Tg4 mice exhibited strong RFP expression, with the RFP signal stronger in Tg3 than in Tg4 mice (Fig 2a). Parallelly, we crossed *Avil-*Cre knock-in mice [23] and Rosa26-loxP-stop-loxP-nls-LacZ (RNZ) reporter mice [24] and obtained *Avil-*Cre:RNZ mice, which were used for X-gal staining of the TGs in adulthood (> P45) (Fig 2b). We compared this pattern with the one displayed by RFP^+^ neurons in the TGs of adult *Avil-*nlsRFP mice and found the two to be almost identical. DRGs also exhibited a higher RFP expression in Tg3 than in Tg4 (Fig 2c).

We noticed that a small ratio of Tg3 mouse offsprings showed weaker RFP expression compared to other Tg3 littermates (S2a Fig). To elucidate the cause of this discrepancy, we performed PCR amplification of BAC ends and found that one end of the BAC insert was intact, but the other end was deleted from the chromosome in these mice (Tg3-weak mice) (S2b Fig). Among 59 offspring analyzed from crosses between wildtype females and a Tg3 male backcrossed to ICR 5 times (N5), 27 mice (from 3 litters) had the *RFP* gene. Within these 27 mice, 3 (11%) were found to lack one BAC end and they all showed weak RFP fluorescence. The remaining 24 mice had intact BAC insert and all of them analyzed (18 of 24) exhibited strong RFP fluorescence. Thus, the Tg3 line appeared to be unstable. To obtain Tg3 mice with strong RFP signal, it is necessary to select mice with intact BAC ends, which occupy about 89% of *RFP*-positive Tg3 offsprings. On the other hand, among 17 offsprings from crosses between wildtype females and Tg4 males backcrossed to ICR 5–6 times, 8 mice (from 2 litters) were Tg mice, all of which had both BAC ends intact (S2b Fig).

**Fig 3:**
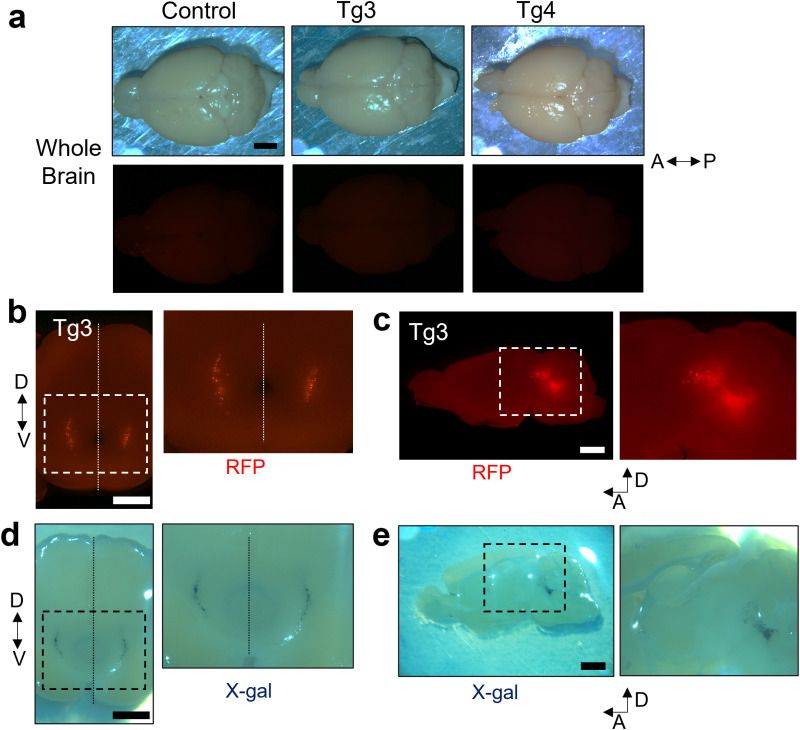
RFP expression in the brain of *Avil-*nlsRFP mice in adulthood. (a) Images of whole brains of adult mice taken from the top (dorsal view). Brains of both *Avil*-nlsRFP Tg3 (n = 4) and Tg4 (n = 3) mice showed no obvious RFP signal and appeared similar to the WT control animal brains (n = 3). Representative brightfield and fluorescent images are shown. A: Anterior, P: Posterior. (b, c) Coronal (b: n = 1) and sagittal (c: n = 1) brain sections (1 mm-thick) from adult *Avil*-nlsRFP Tg3 mice showed a weak RFP signal in a specific midbrain region. Magnified image of the area enclosed by the dashed line is also shown. D: Dorsal, V: Ventral. (d, e) Coronal (d: 400 μm-thick, n = 2) and sagittal (e: 200 μm-thick, n = 2) brain sections from adult *Avil*-Cre:RNZ mice showed X-gal signals in a specific midbrain region. Magnified images of areas enclosed by the dashed lines are also shown. Scales: 1 mm.

We also examined RFP expression in the brains of *Avil-*nlsRFP Tg3 and Tg4 mice. We imaged the whole adult brains along the dorsoventral axis but observed no apparent fluorescence (Fig 3a). Additionally, we made 1 mm-thick coronal and sagittal sections from the adult Tg3 mice to systematically examine RFP expression across different brain regions. In coronal sections, the RFP signal was observed in a specific part of the midbrain (Fig 3b) and was confirmed in sagittal sections (Fig 3c). Parallelly, we performed X-Gal staining in 400 μm-thick coronal and 200 μm-thick sagittal brain sections from adult *Avil-*Cre:RNZ mice to visualize the *Avil*^+^ regions. LacZ activity was observed in areas similar to where the RFP signal was detected in *Avil-*nlsRFP Tg3 mice brain sections (Fig 3d and 3e). We also checked the RFP signal in the heart, lung, kidney, liver, hind-leg muscle, stomach, intestine, and testis of adult *Avil-*nlsRFP Tg3 mice and found no evident fluorescence (S3 Fig). This result was consistent with a previous study that found no LacZ activity in the liver, skin, thymus, testis, heart, and ovaries of *Avil-*Cre:LacZ reporter mice [17]. Overall, the *Avil-*dependent expression pattern in the adult *Avil-*nlsRFP Tg3 mice was almost identical to that of adult *Avil-*Cre knock-in mice.

**Fig 4:**
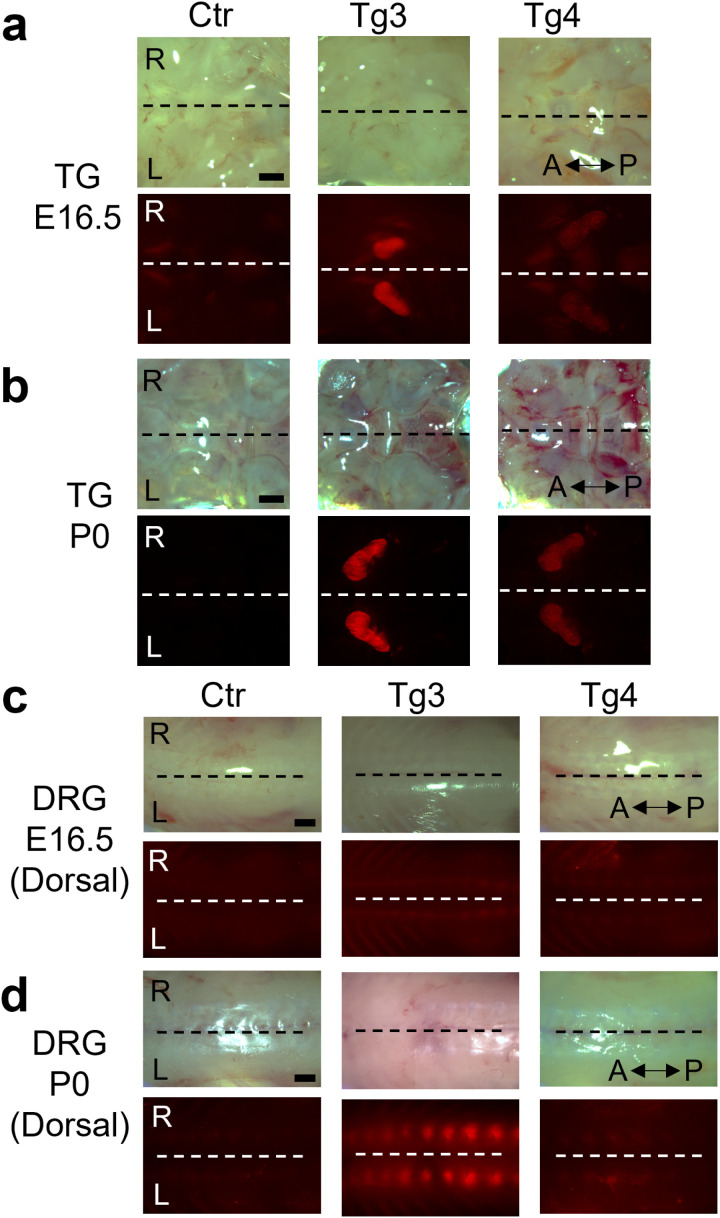
RFP expression in TG and DRG of *Avil-*nlsRFP Tg mice across perinatal stages. The RFP signal was assessed in the TG and DRG of Tg3 and Tg4 mice at E16.5 and P0 to elucidate the expression across perinatal period. Representative brightfield and fluorescent images are shown. (a, b) Representative images of whole TG attached to the cranial base at E16.5 (a) and P0 (b). The RFP signal was strong for Tg3 pups, but weaker for Tg4 pups. (c, d) Representative images of whole DRG (dorsal view) embedded along the spinal cord at E16.5 (c) and P0 (d). Both Tg3 and Tg4 animals showed no obvious RFP signal at E16.5 stage and appeared similar to control. By the P0 stage, the RFP signal became stronger and more obvious for Tg3 pups but continued to be weak for Tg4 animals. [n = 6 (Control, E16.5), n = 3 (Tg3, E16.5), n = 5 (Tg4, E16.5), n = 7 (Control, P0), n = 4 (Tg3, P0 and Tg4, P0)]. A: Anterior, P: Posterior, R: Right, L: Left, Ctr: Control, dashed lines: midlines. Scale bars: 1 mm.

Next, we compared RFP expression in TGs and DRGs from embryonic development to birth. We imaged whole embryos at E12.5, E13.5, and E14.5 stages, and imaged TGs and DRGs isolated from E16.5 embryos and P0 pups. We did not detect any obvious RFP expression in the whole embryos until E14.5. At the E16.5 and P0 stages, the TGs of Tg3 mice exhibited a bright RFP signal; however, the expression at P0 was visibly stronger than that at E16.5 (Fig 4a and 4b). In Tg4, the TGs displayed very weak RFP expression at E16.5, which became stronger by P0 (Fig 4a and 4b). We also examined DRG embedded along the spinal column from the dorsal side and found that Tg3 and Tg4 embryos had a very weak RFP expression at E16.5, making it difficult to identify the organ based on fluorescence intensity (Fig 4c). By P0, RFP expression in DRG of Tg3 pups significantly improved, and the organ could easily be identified (Fig 4d). In DRG of Tg4 mice, even at P0, the RFP expression was very weak, and the organ remained difficult to visualize (Fig 4d). A previous study by our group compared RFP expression in TG of *Avil-*nlsRFP Tg3 mice at P0, P4–P6, P14–P16, and > P60 stages and reported that the RFP signal did not increase in strength from P0 to adult stages [15]. Thus, in *Avil-*nlsRFP Tg3 mice, the *Avil*-RFP expression in the TG and DRG conspicuously increases in intensity until P0 and remains strong throughout P4-6, P14-P16, and adult stages.

**Fig 5:**
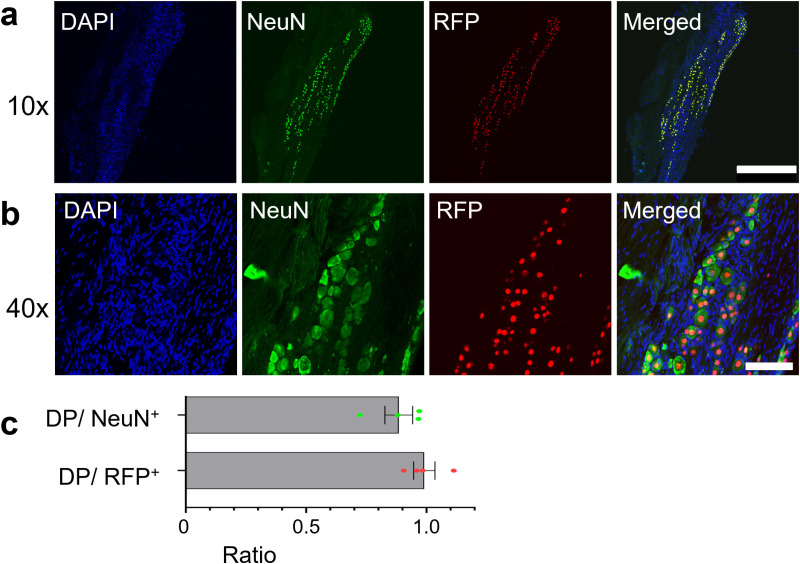
Co-localization of RFP and NeuN signals in TG of adult *Avil-*nlsRFP Tg3 mice. (a, b) Confocal images of adult *Avil*-nlsRFP Tg3 TG sections (RFP: Red) stained for an NeuN antibody (Green), and DAPI (Blue). Representative images are shown at 10 x (a) and 40 x (b) magnifications. Scale bars: 500 μm (a), 100 μm (b). (c) Horizontal bar graphs demonstrate the ratio of double positive cells (DP) to NeuN^+^ and RFP^+^ cells as labelled. Each dot represents an individual section. Error bars: SEM. [n = 4 (2 sections each from 2 animals)].

Lastly, we aimed to evaluate the ratio of peripheral sensory neurons expressing RFP in *Avil-*nlsRFP Tg3 mice We made sections of adult TG and labeled the ubiquitous cell population using DAPI, and neurons using a neuron-specific antibody (NeuN) (Fig 5a and 5b). We found that 99.1% ± 4.4% of RFP^+^ cells were positive for NeuN, indicating that *Avil*-nlsRFP expression is highly specific to neurons. Additionally, 88.5% ± 5.8% of NeuN^+^ cells were positive for RFP, indicating that almost all neurons expressed RFP (Fig 5c). This result is consistent with previous studies that observed ~82%–100% of neurons in TG and DRG to be *Avil*-positive [8,17–19].

Our generated *Avil-*nlsRFP Tg mouse lines expressed RFP in almost all peripheral sensory neurons (Figs 2, 4 and 5). Some brain regions displayed a mild RFP signal (Fig 3). These results were consistent with previous reports of *Avil* expression in the brain [8,17]. Based on our findings, we recommend using Tg3 mice for studying the TG across embryonic and postnatal stages and DRG only across the postnatal stages. To maintain Tg3 line of mice expressing strong RFP signal, it will be necessary to consistently exclude the small population (~11%) of Tg offsprings in which one of the BAC ends is deleted from the chromosome (Tg3-weak mice) (S2b Fig). Tg4 mice may be valuable for studying TG across the postnatal stages and DRG in the adult stage. Since their RFP expression is linked to the Y chromosome, the Tg4 pups can be identified based on gender alone without genotyping, making it easier to use. Overall, our *Avil-*nlsRFP Tg mouse lines will help visualize sensory ganglia in mice easily. This tool is advantageous throughout development, especially during the embryonic and early postnatal stages when the ganglia are challenging to distinguish and demarcate from their surrounding tissues. For *in vivo* studies, this tool would ease the job of researchers performing survival surgeries/procedures involving the TG or DRG in mice by making the organ convenient to identify under a fluorescent filter. It would be equally beneficial for researchers wanting to isolate the sensory ganglia across developmental stages.

Furthermore, this tool will help immunohistology studies in TG and DRG sections since the neuronal nuclei will already be RFP-labelled. Additionally, since our promoter is sensory neuron-specific, our tool would be valuable for researchers wanting to isolate specifically the neurons from the heterogeneous cell population in the ganglia by fluorescence-activated cell sorting. However, this mouse line presents a limitation. Since only the sensory neuronal nuclei are RFP-labelled, visualizing the cell membrane and sensory neuronal afferents will be impossible: thus, it may not be useful for soma and axonal morphologies studies. In conclusion, *Avil-*nlsRFP mice are valuable for visualizing and identifying the sensory ganglia and peripheral sensory neurons across developmental stages. We hope this biological resource can act as a helpful tool for further research in somatosensation.

## Materials and methods

### Animals

All experiments were performed according to the guidelines for animal experimentation of the National Institute of Genetics (NIG) and were approved by the animal experimentation committee of the NIG. Our study is in accordance with the ARRIVE guidelines. Sex of pups was not identified. *Avil-*Cre (RBRC10246) [23] and R26-LSL-nls-LacZ (RNZ) (RBRC02657) [24] mice were previously reported.

### Generation of *Avil-*nlsRFP BAC Tg mouse

To obtain pK358.nlsRFP-WPRE-pA-FRT-Amp-FRT, the nlsTurboRFP (nlsRFP) sequence was amplified from pK071.CAG-Cre-ires-nlsRFP-WPRE using the Platinum Pfx DNA polymerase (Invitrogen, #45−0031) and KS141/KS142 primers, and blunt-end ligated with the EcoRV-digested pK296. The orientation of the insert within the pK358 was confirmed by sequencing. The nlsRFP sequence in pK071 was obtained by PCR amplification of pK025.CAG-RFP [25] using Platinum Pfx enzyme and the primer pair WL005 and HM30. To obtain pK296.WPRE-pA-FRT-Amp-FRT, pK295 was digested with SpeI and treated with shrimp alkaline phosphatase (TaKaRa #2660A) to ligate with the WPRE-pA cassette. To obtain pK295, the NotI site was removed from the pK294.FRT-Amp-FRT (pS120) vector [22] by double digestion with EcoRV/XhoI, followed by self-ligation. To obtain the WPRE-pA cassette, the WPRE-pA sequence was amplified from pK187.GAP43-GCaMP6s-WPRE-pA by PCR using the KOD-Plus DNA polymerase (Toyobo) and KS136/PB01 primers, inserted into the Topo Blunt IV vector (Invitrogen, #45−0031) and excised by SpeI digestion. WPRE and pA sequences in pK187 were derived from pK029 [25] and pK047.GAP43-EGFP-pA [26], respectively.

The bacterial artificial chromosome (BAC) Tg construct was generated using Red/ET system (Gene Bridges) as described [22]. The 175-kb BAC clone (RP23-109K12), which contains the mouse Advillin locus, was used. The nlsRFP-WPRE-pA-FRT-Amp-FRT sequence in pK358 was amplified by PCR with the Platinum Pfx DNA polymerase and primer pair KS140 (F-cassette) and KS139 (R-cassette) and was processed for BAC recombination. The FRT-Amp-FRT sequence in the modified BAC construct was removed by flpe/FRT recombination in bacteria. The final BAC Tg construct was linearized by digestion with PI-Sce1 (BioLabs, #R0696) and purified using a Sepharose column. Fractions #5-#9 were examined by pulsed-field gel (PFG) electrophoresis in 1% agarose (SeaKem Gold) in 0.5 x TBE at 14°C for 16 h (5V/cm, Switch Time 50 s to 90 s, FIGE Mapper: Bio-Rad). A lambda ladder PFG marker ranging from 48.5 kb to 1018.5 kb (L: BioLabs N0340S) was used. Fraction #7, which contained the largest amount of the BAC construct, was diluted to 1 ng/μl in 10 mM Tris (7.5), 0.1mM EDTA, 100 mM NaCl, and microinjected into the fertilized eggs of B6C3 F2 mice. Among 4 founders (#1, #2, #3 and #4) obtained, founders #1, #3, #4 were backcrossed with wildtype ICR mice to create transgenic lines #1, #3 and #4 (Tg1, Tg3 and Tg4). The progeny was analyzed for BAC insertion by PCR amplification with the primers: KS149 & KS150 (~500 bp band for *Avil*-nlsRFP^+^ mice, and no band for wildtype mice). Tg3 and Tg4 mice that were backcrossed to ICR 3–6 times were used for all experiments in the current study. KS413/KS414 and KS415/KS416 PCR primer pairs from the BAC vector were used to check whether full length of Tg construct was integrated into the chromosome in Tg3 and Tg4 mice [27]. The combination of KS413/KS414 and KS421/KS161 primer pairs was used to distinguish Tg3 and Tg3-weak mice (See S2b Fig right).

### Oligos

**Table:** 

Primer ID	Primer Sequences (5’ to 3’)
KS157 (*Avil* Cre)	CCCTGTTCACTGTGAGTAGG
KS158 (*Avil* Cre)	AGTATCTGGTAGGTGCTTCCAG
KS159 (*Avil* Cre)	TGTTTCACTATCCAGGTTACGGA
KS136 (F_Spe1_EcoRV_WPRE)	CGCACTAGTGATATCAATTCGCCCTTCGGCC
KS149 (*Avil*-nlsRFP)	GGCACCCAGACCATGAAGAT
KS150 (*Avil*-nlsRFP)	CCAGTTTGCTAGGGAGGTCG
KS141 (XhoI_nls_F)	CTCGAGACCATGGGCCCAAA
KS142 (A-TturboRFP_R)	ATCATCTGTGCCCCAGTTTGC
KS139 (R_Avil_FRT)	CCTCAATTCTCCATGTGATGATCCGGGGGTCGTTGCTCACAGCCCTGAAGGCACTGCCTTGACCAAGTTGCTGAAG
KS140 (F_Avil_nls)	TGAGTAGGATCACCCCGACTTTGTGATGTTTCAGTTCCAGGAAGACAGCCACTATGGGCCCAAAGAAGAAGAGAAAG
KS413 (pBAC Ce3.6 BAC1_F)	ACAGCAGCAAAACGAAAAAT
KS414 (pBAC Ce3.6 BAC1_R)	CTGAACGTTCTGATATGTTT
KS415 (pBAC Ce3.6 BAC2_F)	GCGCGCCAATAGTCATGC
KS416 (pBAC Ce3.6 BAC2_R)	GCCGCAAATTTATTAGAGCA
KS421 (TurboRFP_F II)	CAACACCGAGATGCTGTACC
KS161 (TurboRFP_R)	CCAGTTTGCTAGGGAGGTCG
WL005 (XhoI-ntRFP-F)	GCTCGAGACCATGGGCCCAAAGAAGAAGAGAAAGGTTTCGAGCGAGCTGATCAAG
HM30 (Rc-RFP-Not1)	GTCGCGGCCGCATCATCTGTGCCCCAGTTT
PB01 (Reverse_SpeI_polyA)	CTGACTAGTTTAAGATACATTGATG

### X-Gal staining

*Avil-*Cre:RNZ mice were obtained by mating *Avil-*Cre males with homozygous RNZ females. PCR primers used for genotyping of *Avil-*Cre mice were KS157/KS158/KS159. (~1.5 kb and 3.5 kb bands for WT and KI alleles, respectively). Adult (>P45) *Avil-*Cre:RNZ mice were anaesthetized with pentobarbital intraperitoneal injection and perfused with 0.9% NaCl. Brains and TGs were isolated and incubated in 4% paraformaldehyde (PFA) in 0.1 M phosphate buffer (PB) (PH 7.4) for 2 h at 4^o^C for fixation. The isolated organs were then rinsed thrice in 0.05 M PB and transferred to 30% sucrose in 0.1 M PB at 4^o^C till sectioning. 400-μm coronal and 200-μm sagittal brain sections were made using a freezing microtome (REM-710, Yamato). Brain sections and TG were rinsed in 0.05 M PB and transferred to X-Gal staining solution (5 mM potassium ferricyanide, 5 mM potassium ferrocyanide, 2 mM magnesium chloride, 1 mg/ml X-Gal in 0.05 M PB) at 37^o^C overnight. The following day, brain sections and TGs were rinsed thrice with 0.05 M PB. Images were acquired using the M205 FCA microscope (Leica) and DFC7000T camera (Leica).

### Immunohistochemistry

Adult *Avil-*nlsRFP Tg3 mice were anesthetized with intraperitoneal injection of pentobarbital and transcardially perfused with saline (0.9% NaCl) followed by the fixative 4% PFA in 0.1M PB. The TG was isolated while still attached to the cranial base and incubated overnight in 4% PFA in 0.1M PB at 4^o^C for post-fixation. The following day, the isolated organ was rinsed with 0.1M PB and transferred to 30% sucrose in 0.1M PB at 4^o^C till sectioning. For sectioning, individual TG was carefully separated from the cranial base and embedded in OCT compound (Sakura Finetek, #4583). 20 μm-thick longitudinal sections were made using a cryostat (Leica CM3050s). Sections were mounted on MAS-coated slides (Matsunami MAS-02) and dried overnight at room temperature. The following day, immunostaining was performed. Firstly, the slides were incubated in blocking buffer (3% goat serum, 0.5% TritonZ-100 in phosphate buffer saline (PBS)) for 2 h at room temperature. Following this, the slides were incubated in primary antibody solution: Anti-NeuN (clone A60) (1:1000, Merck MAB377) overnight on a shaker at 4^o^C. The next day, slides were rinsed with 1x PBS and incubated in secondary antibody solution: Alexa 488 (1:1000, Goat anti-rabbit IgG, Invitrogen #A11034) on shaker for 1 h at room temperature. Following this, the slides were again rinsed with 1x PBS and DAPI solution (1 ug/ml) was applied for 2 min at room temperature in the dark. The slides were again washed with 1x PBS and mounting medium (0.2% n-Propyl gallate in 90% glycerol_1x PBS) was applied followed by a coverslip. The slides were kept at 4^o^C overnight and sealed the following day. Images were acquired using a confocal microscope (Leica TCS SP5 II) and a 10x and 40x lens. Individual cells in images acquired at 40x magnification were manually counted using Fiji ImageJ.

### Imaging RFP signal from TG, DRG, brain, and other organs

Adult mice were anesthetized with pentobarbital intraperitoneal injection and perfused with 0.9% NaCl followed by 4% PFA in 0.1 M PB for fixation. The whole brain, TG, DRG, and other organs of interest were harvested and rinsed in 0.1M PB. P0-P1 and P6-P8 pups were decapitated, and organs of interest were harvested and rinsed with 0.1M PB. For imaging the embryos during various stages of development, the pregnant mother was sacrificed by cervical dislocation. Embryos were extracted from the uterus and rinsed with 0.1 M PB. Till E13.5 stage, images of whole embryos were acquired to evaluate RFP signal. E14.5 onwards, TG and DRG were harvested from the embryos and imaged separately.

All Images were acquired under the brightfield and fluorescent filter using a M205 FCA microscope (Leica) and DFC7000T camera (Leica).

For the purpose of sectioning, the adult mouse brains were transferred to 30% sucrose in 0.1 M PB at 4^o^C overnight. Brain matrix (Alto #68-1275-1, ASI #RBM-2000C) was used to make 1 mm coronal and sagittal sections. A freezing microtome was used to make 400-μm coronal and 200-μm sagittal sections.

### Statistical analysis and computing

Fiji/ImageJ ver. 1.53f51 and GraphPad Prism version 9.3.1 were used for data analysis and visualization. Unless otherwise mentioned, data are presented as mean ± standard error of mean (SEM). Sample size for all the results is described in the Figure legends.

## Supporting information

S1 Fig RFP signals in TGs of three lines of *Avil-*nlsRFP mice. Top view images of the whole trigeminal ganglia (TGs) attached to the cranial base at postnatal day (P)6-P8. All of Tg1, Tg3 and Tg4 mice showed RFP expression in the TG. However, RFP signal in Tg1 mice (n = 2 mice) was much weaker than that in Tg3 (n = 2 mice) and Tg4 (n = 2 mice) mice. Bright field and RFP images of a wildtype (WT) littermate of Tg1 pup are shown as control (Ctr). R: right; L: Left; A: apical; P: postal. Scale: 1 mm.
(TIF)

S2 Fig Occasional appearance of weak RFP expressing mice in the *Avil-*nlsRFP Tg3 line. (a) Top view images of the whole TGs in adulthood. In crosses between Tg3 and WT mice, Tg3-weak mice, which show weaker RFP signal in the TG than Tg3 and Tg4, were occasionally found. Scale: 1 mm. (b) In Tg3-weak mice, one end of the insert (BAC Tg construct) was present but the other end was absent in the chromosome, suggesting that the BAC insert was partially deleted from the chromosome. On the other hand, Tg3 and Tg4 mice had the intact insert. (Left) The PCR primer pair (KS415/ KS416) was used to amplify one of the BAC ends (116-bp). (Right) A mixture of two PCR primer pairs (KS413/ KS414 and KS421/KS161) were used to amplify the other BAC end (520-bp) and the *RFP* gene (260-bp), respectively. PCR products from two each of Tg3, Tg3-weak and Tg4 mice and a WT mouse were loaded on 2% agarose gel in TAE with a size marker.
(TIF)

S3 Fig Analysis of RFP signals in various organs of *Avil-*nlsRFP Tg3 mice. Indicated organs were collected from the same Tg3 and control (Ctr) mice used in Fig 2a. No evident RFP fluorescence was detected in the heart, lung, liver, kidney, hind-leg muscle, stomach, intestine, and testis of adult *Avil-*nlsRFP Tg3 mice (n = 2). Scales: 1 mm.
(TIF)
