# Supplementary figures and images for: Generation and characterization of BAC transgenic mouse lines expressing a fluorescent protein in trigeminal and dorsal root ganglion neurons
Source: PLoS One. 2025 Jun 6;20(6):e0321014. doi: 10.1371/journal.pone.0321014 (PMC12143492; doi:10.1371/journal.pone.0321014)

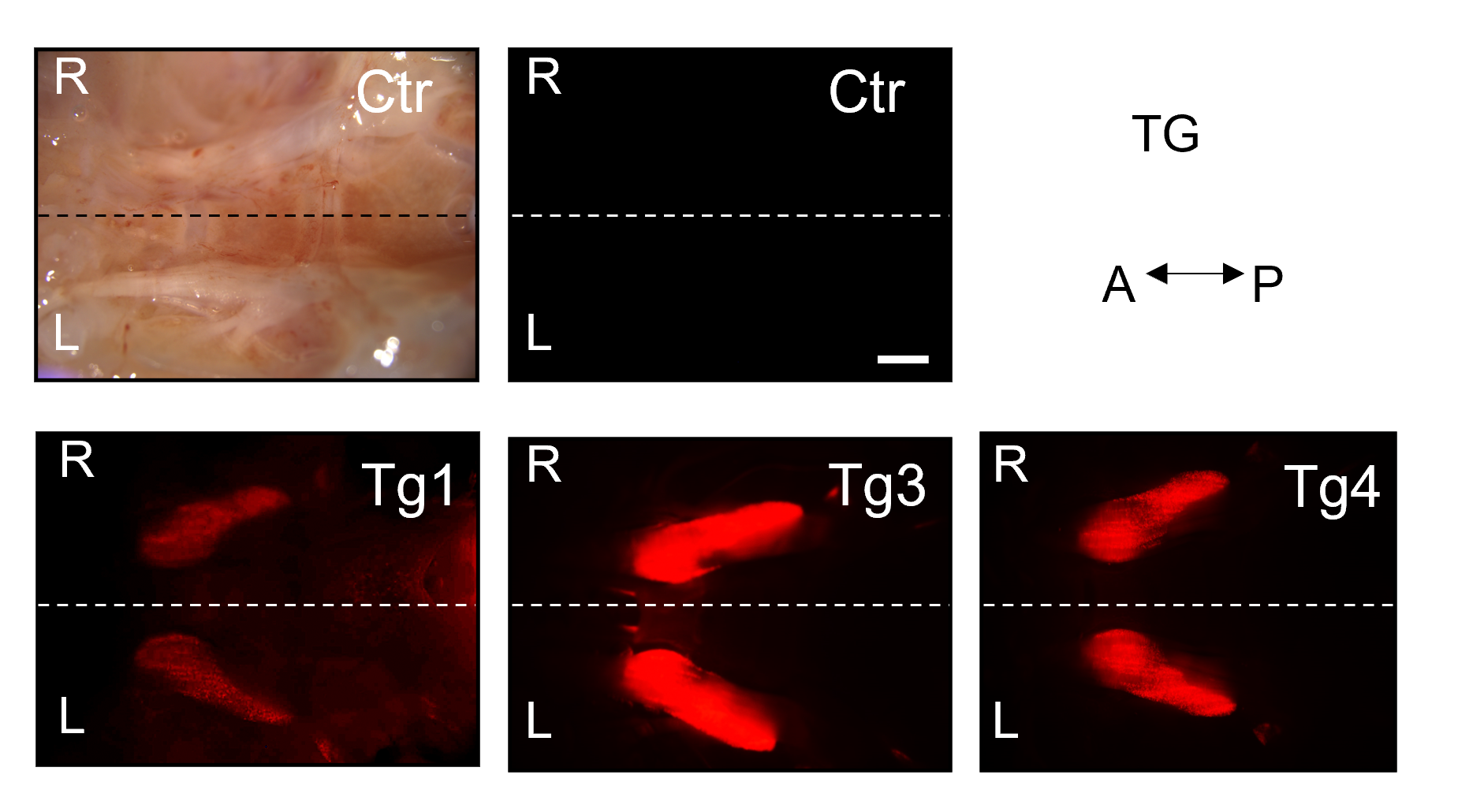

Supplement: S1 Fig — Top view images of the whole trigeminal ganglia (TGs) attached to the cranial base at postnatal day (P)6-P8. All of Tg1, Tg3 and Tg4 mice showed RFP expression in the TG. However, RFP signal in Tg1 mice (n = 2 mice) was much weaker than that in Tg3 (n = 2 mice) and Tg4 (n = 2 mice) mice. Bright field and RFP images of a wildtype (WT) littermate of Tg1 pup are shown as control (Ctr). R: right; L: Left; A: apical; P: postal. Scale: 1 mm. (TIF) [file pone.0321014.s001.tif]

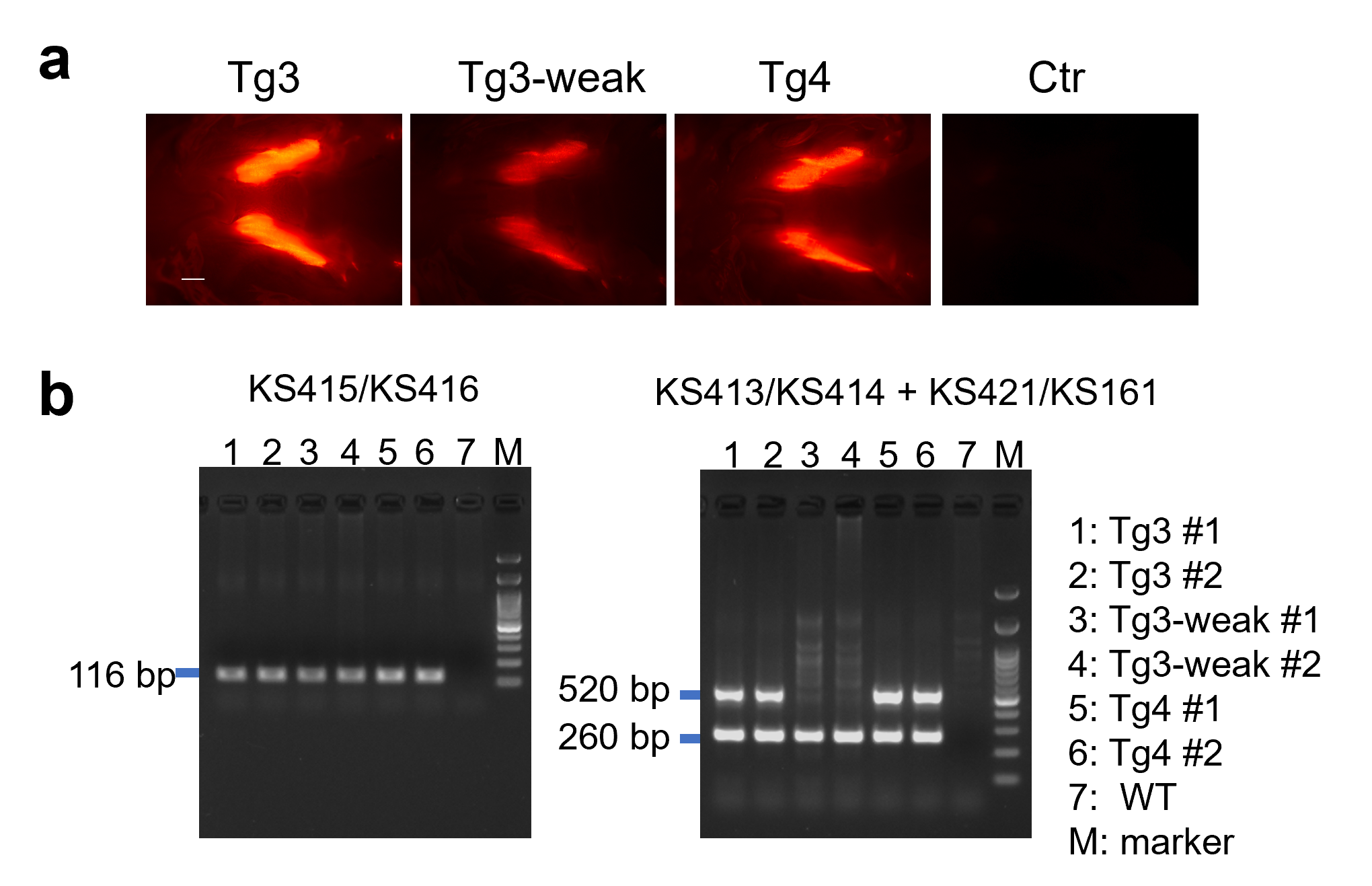

Supplement: S2 Fig — (a) Top view images of the whole TGs in adulthood. In crosses between Tg3 and WT mice, Tg3-weak mice, which show weaker RFP signal in the TG than Tg3 and Tg4, were occasionally found. Scale: 1 mm. (b) In Tg3-weak mice, one end of the insert (BAC Tg construct) was present but the other end was absent in the chromosome, suggesting that the BAC insert was partially deleted from the chromosome. On the other hand, Tg3 and Tg4 mice had the intact insert. (Left) The PCR primer pair (KS415/ KS416) was used to amplify one of the BAC ends (116-bp). (Right) A mixture of two PCR primer pairs (KS413/ KS414 and KS421/KS161) were used to amplify the other BAC end (520-bp) and the RFP gene (260-bp), respectively. PCR products from two each of Tg3, Tg3-weak and Tg4 mice and a WT mouse were loaded on 2% agarose gel in TAE with a size marker. (TIF) [file pone.0321014.s002.tif]

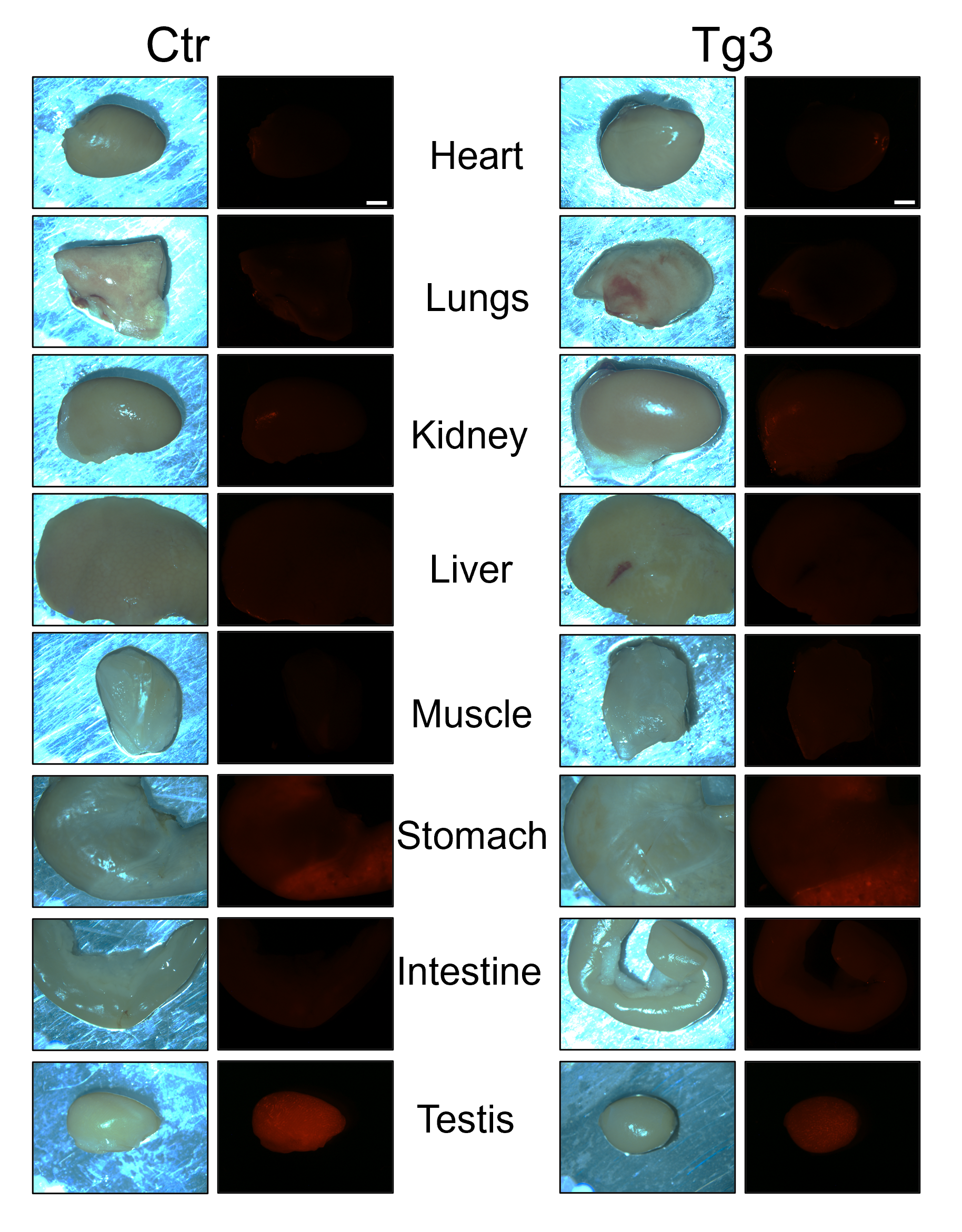

Supplement: S3 Fig — Indicated organs were collected from the same Tg3 and control (Ctr) mice used in Fig 2a. No evident RFP fluorescence was detected in the heart, lung, liver, kidney, hind-leg muscle, stomach, intestine, and testis of adult Avil-nlsRFP Tg3 mice (n = 2). Scales: 1 mm. (TIF) [file pone.0321014.s003.tif]
